# Comparison of Kinetic Study and Protective Effects of Biological Dipeptide and Two Porphyrin Derivatives on Metal Cytotoxicity Toward Human Lymphocytes

**Published:** 2017

**Authors:** Maryam Khosravi, Rahmatollah Rahimi, Jalal pourahmad, Mohammad Hadi Zarei, Mahboubeh Rabbani

**Affiliations:** a *Bionorganic Chemistry Research Laboratory, Chemistry Department, Iran University of Science and Technology, Narmak, Tehran, Iran. *; b *School of Pharmacy, Pharmaceutical Sciences Research Center, Shahid Beheshti University of Medical Sciences, Tehran, Iran.*

**Keywords:** Dipeptide, Porphyrin, Chelator, Toxic metals, Cytotoxicity, EC_50_, lymphocytes

## Abstract

In this research, dipeptide (his-*β*-alanine) and porphyrin derivatives were choosen for comparing chelating ability of toxic metals such as Al^3+^, Cu^2+^, Hg^2+^ and Pb^2^^+ ^*in-vitro*. The reason for choosing these two compounds is that both of them are naturally present in biological systems and comparison of chelating ability of these two compounds has not yet been done. Synthesis and comparison of kinetic study of dipeptide (his-*β*-alanine), meso-tetrakis(4-trimethylanilinium) porphyrin (TAPP) and Tetrakis(4-sulfonatophenyl)porphyrin (TPPS_4_) were carried out by our team. In addition, cytotoxicity assays of metals and chelators were also performed using methylthiazoletetrazolium (MTT) test. Furthermore we investigated the protective effect of chelators against cytotoxicity, induced by differenrt toxic metals such as Al^3+^, Cu^2+^, Hg^2+^ and Pb^2+ ^on human lymphocytes. EC_50_ values on human lymphocytes obtained after 12 h. incubation for Al^3+^, Cu^2+^ and Hg^2+ ^were 30, 51, 3 µM respectively and for Pb^2+^ no cytotoxicity was observed on human lymphocyte up to 1000 µM concentration. EC_50_ obtained for chelators dipeptide, TPPS_4_ and TAPP were 948, 472 and 175 µM respectively. Pretreatment of human lymphocyte with subtoxic concentations of chelators reduced toxicity of the metals against human blood lymphocytes.

## Introduction

In this reserch, peptide and porphyrin derivatives were choosen for comparison, because all of them possess important roles in biological systems (1-9). Peptides are one of the best options for drug development due to their high specificity and low toxicity. Peptides are mostly synthesised by biological technology or chemical methods. The chemical method, mainly solid-phase peptide synthesis (SPPS) is usually used for high production because of its simplified reaction and ordinary purification/isolation steps for the target products (10-13). Porphyrins are a group of tetrapyrrole pigments. Physical and chemical properties of porphyrins are often related to their compositions and structures.The biological role of porphyrins has been known since discovery of significantly biological macromolecules like hemoglobin and chlorophyll. The porphyrins have been applied at different fields such as photo converter, photo catalyst, photo sensitizerin theraputics, diagnosis of diseases and also photo dynamic therapy and anti-Aids (HIV) drug(14-18). Complexation of peptides and porphyrins with numerous cations, their cytotoxicity and EC_50_ concentrations have so far been studied *in*-*vitro* and *viv*o (19-31). In this paper, we reported synthesis of dipeptide (his-*β*-alanine) meso-tetrakis (4-trimethylanilinium) porphyrin (TAPP) and Tetrakis (4-sulfonatophenyl) porphyrin (TPPS_4 _) as chelating agents for chelating of toxic metals such as Al^3+^, Cu^2+^, Hg^2+^ and Pb^2+^and compared their kineticsby UV-Visible spectrophoto meteric methods. We also determined molecular structure of the dipeptide and porphyrin compounds using different methods such as UV-Visible spectrophotometry, FT-IR, ^1^H NMR and LC-Mass spectrometry only for dipeptide structure. Furthermore, *in-vitro* cytotoxicity assay was done using MTT test and EC_50_ values for metals and chelators on human lymphocyte were obtained after 12 h. incubation. lymphocyte has fundamental roles in the immune system because they are the cells that determine the specificity of the immune response to infectious microorganisms and other foreign substances. Finally we studied protective effect of our synthesized chelators against cytotoxicity of metals on human lymphocyte and results clearly indicated that the chelators reduced toxicity of applied metals in this cellular model.

## Experimental

*Materials*

*Chemicals for dipeptide synthesise*: Wang resin, N-Fmoc-N-trityl-L-histidine were purchased from Bachem chemical Company. Boc-β-Alanine-OH was obtained from Aldrich Company, Hydroxybenzotriazole (HOBt) purchased from Fulka Company. Scavengers (anisole andphenol) solvents (trifluoroacetic acid (TFA) piperazine, N,N′-Diisopropylcarbodiimide(DIC) diethyl ether, dichloromethane, N,N-dimethyl formamide and methanol, Acetic anhydride, pyridine were obtained from Merck chemical Company *Chemicals for porphyrin synthesise:* Propionic acid, dimethylaminobenzaldehyde, benzaldehyde, pyrrole, sulfuric acid, acetone**, **sodium carbonate**, **methyl iodide and amberlite resin were obtained from Merck chemical Company.

*Chemicals for investigation of cytotoxicity*

3-(4,5-Dimethyl-2-thiazolyl) 2,5-diphenyl-2H-tetrazolium bromide (MTT) was purchased from Sigma-Aldrich Company, Ficoll-paque PLUS was obtained from Ge Healthcare Bio-Science Company. RPMI1640 and FBS Fetal Bovine serum) were purchased from Gibco, Life Technologies, Grand Island, NY.The metal salts AlCl_3_, CuCl_2_, HgCl_2_ and Pb(NO_3_)_2_ were obtained from Merck chemical Company.

*Sample characterization*

PG Instruments T80 Double Beam UV-Visible spectrophotometer was used for UV-Visible measurements. The infrared spectra were recorded on a Shimadzu FT-IR-8400S spectrophotometer in the range of 400–4000 cm^−1^. Proton NMR spectra were recorded on a Bruker DRX250 (300 MHz) spectrometer in water. LC-MS analyses were performed on Agilent 6410 Triple Quadruple LC-MS (USA).

*Dipeptide synthesis*

**Scheme 1:**
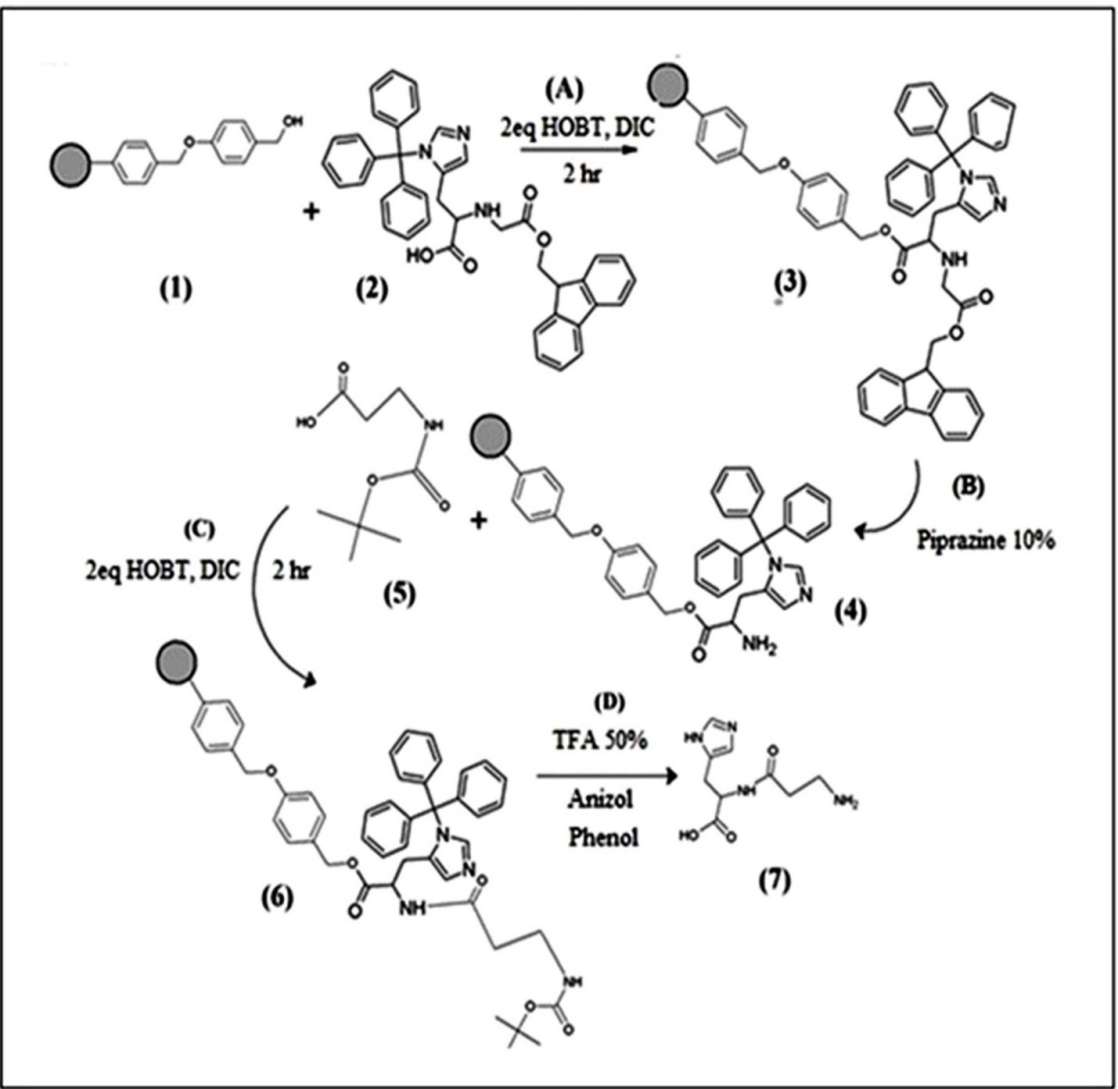
Steps of dipeptide(his-*β*-alanine) synthesis

Dipeptide (his-*β*-alanine**)** was manually synthesized on solid phase using standard Fmoc and Boc Strategy (Scheme1) (32, 33)**. **Briefly, the peptide sequence (his-*β*-alanine) was assembled on wang resin (1). *N*-Fmoc-*N*-trityl-*L*-histidine (2 equivalents) (2) and two equivalents (HOBt) and (DIC) as a coupling reagent were added to the reaction vessel. The mixture was shaked for 2 h. After, first amino acid is loaded to the resin the un-reacted sites must be end-capped with 2 equivalents acetic anhydride and 2 equivalents pyridine to ensure that future reactions do not react at those unloaded sites. Removal of the Fmoc group (3) was done by the addition of 10% Piperazine, shacked for 30 min under nitrogen atmosphere. The monitoring of the completion of the Fmoc cleavage, performed with colordetector (acetaldehyde/chloranil) for detection of free terminal amino groups (4) (34). Sec amino acid Boc-*β*-alanine-OH (2 equivalents) (5) treated with coupling reagents (HOBt) and (DIC), which added to the resin and shook for 1.5 h at room temperature. In the last step of the synthesis, peptide was cleaved from the resin, the solvents evaporated and peptide was precipitated with diethyl ether the cleavage of peptide from resin was done using cocktail TFA/TIS/H_2_O/ (82.5:5.5:2.5 v/v) for 45 min. The identity of peptide was confirmed by LC–MS.

*Synthesise of meso-tetrakis(4-trimethylanilinium)porphyrin (TAPP)*

**Scheme 2:**
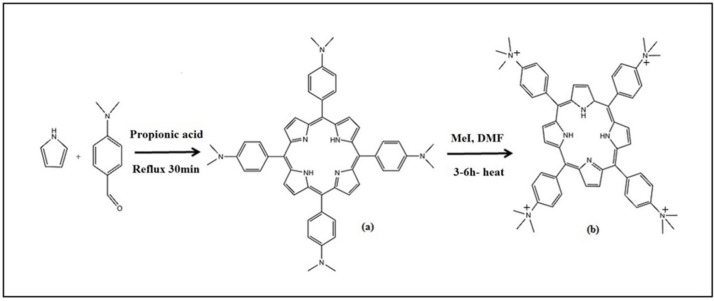
Steps ofmeso-tetrakis (4-trimethylanilinium) porphyrin (TAPP) synthesis

Tetra-(p-dimethylaminophenyl) porphine (a) was synthesized by the method of krishnamurti (Scheme 2). A slurry of tetra- (p-dimethylaminophenyl) porphine (0.87 g) and methyl iodide (5 mL) was heated to reflux for 30 min. Then it was cooled and washed with acetone and dried in vacuume. For preparing perchlorate salt of (TAPP) (b) passing a solution of (TAPP) iodide through a column of anion exchange resin (35).

*Synthesise of Tetrakis(4-sulfonatophenyl)porphyrin(TPPS*_4_ *)*

**Scheme 3:**
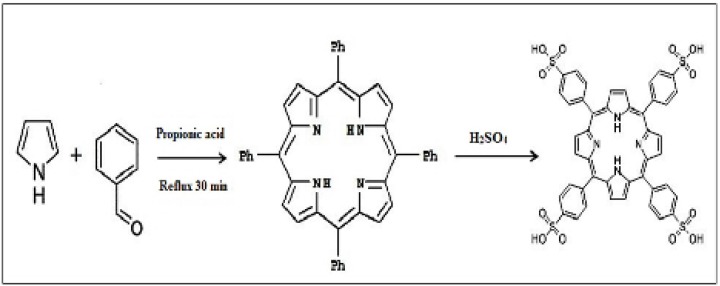
Steps ofTetrakis(4-sulfonatophenyl)porphyrin (TPPS_4_) synthesis

TPPS_4_ was prepared according to the Adler method (Scheme 3). First tetraphenylporphine (36) was synthesized, then sulfonated (35).

*Optimization of concentration of metal saltsforkinitic study*

Different concenterations of metal salts: AlCl_3_, CuCl_2_, HgCl_2_ and Pb(NO_3_)_2_,(10^-1^, 10^-2^, 10^-3^, 10^-4^ and 10^-5^M) were prepered to study the reaction kinetics with chelators [dipeptide (histidine-*β*-alanine), TAPP and TPPS_4_]_. _The changes of absorption in wavelength of 214 nm (maximum wavelength of dipeptide), 416 nm (maximum wavelength of TAPP) and 412 nm (maximum wavelength of TPPS_4_) were investigated by UV-Visible spectrophotometer while concentration of chelators were kept constant at ~10^-5^M vs. metal ions. The kinetic study was done using kinitic mode of spectrophotometr with maximum wavelength of chelators.

*Extraction of lymphocyte from human blood forcytotoxicity study in-vitro*

Blood was obtained from healthy, non-smoking volunteers between 20-30 year old. Isolation of lymphocytes from human blood was done by centrifugation according to standard procedures.And lymphocytes were cultured at concentration of 1×10^4 ^cellsper well in 96-well microplates.

*Cytotoxicity study of metals and chelatorson human lymphocytes in-vitro*

Lymphocytes were cultured in 96-well micro plates and treated with different concenterations of metals and chelators.Cells viability measured after 12 h. incubation by MTT assay. The EC_50_ values for each metals and chelators were determined.

*Effect of dipeptide and porphyrinson metal cytotoxicity toward lymphocyte*

Lymphocyts were treated with EC_50_ values of chelators and kept for 30 min at room temperature. After passing of this time, cells were treated by EC_50_ and 2EC_50_ values of metals. The Cells viability was measured after 12 h. incubation by MTT assay.

*Statistical analysis*

Data were analyzed using one way analysis of variance followed by post hoc Tukey test with GraphPad Prism 5 (Graphpad Software, La Jolla, CA). Results are shown as the mean ± standard error of the mean and P< 0.05 was statistically significant report. For analysis of data at least three independent experiments were used.

## Results and Discussion

*Characterization of synthesised dipeptide (Histidine-β-Alanine)*

Dipeptide was synthesised via the standard BOC method (33). The synthesis of dipeptide (histidine-*β*-alanine) was structurally confirmed by UV-Visible, FT-IR, ^1^H NMR and LC-Mass techniques. The UV-Visible absorbance spectra of Histidine-β-Alanine was obtained in water at 25 °C, the UV absorptions appeared at 214 and 268 nm which can be related to electronic transitions of π→π* and n→π* respectively. The following spectral data for dipeptide was obtained, from the FT- IR spectra (KBr, cm^-1^) with ν_max_: 3238 (NH_2_), 2613-3300(OH) 1643 (N-C=O), 1564 (-C=N), and from ^1^H NMR spectra (300 MHz, D_2_O, δ)*: *7.48 (imidazole ring); 4.69 (2H, CH_2_N) 4.24 (d, CO_2_H). The LC–MS analysis revealed a single mass peak in [M+H]^+^ and [M]^-^ which corresponds to the calculated molecular weight of dipeptide, C_9_H_14_N_4_O_3_, calculated: 226.23, found: *m*/*z*(M+H)^+^: 227.000 and *m*/*z*(M) 224.800. For all above data, please refer to the supplementary information.

**Figure 1:**
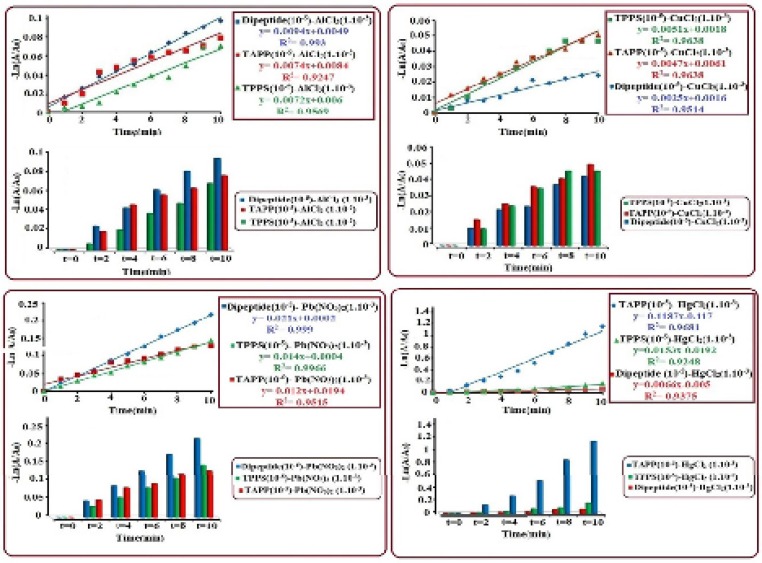
Kinetics study of chelating reactions of dipeptide (histidine-*β*-Alanine), TAPP and TPPS_4_ (10^5-^M) with AlCl_3_, CuCl_2_, Pb(NO_3_)_2_ and HgCl_2_ (10^-3^M

**Figure 2:**
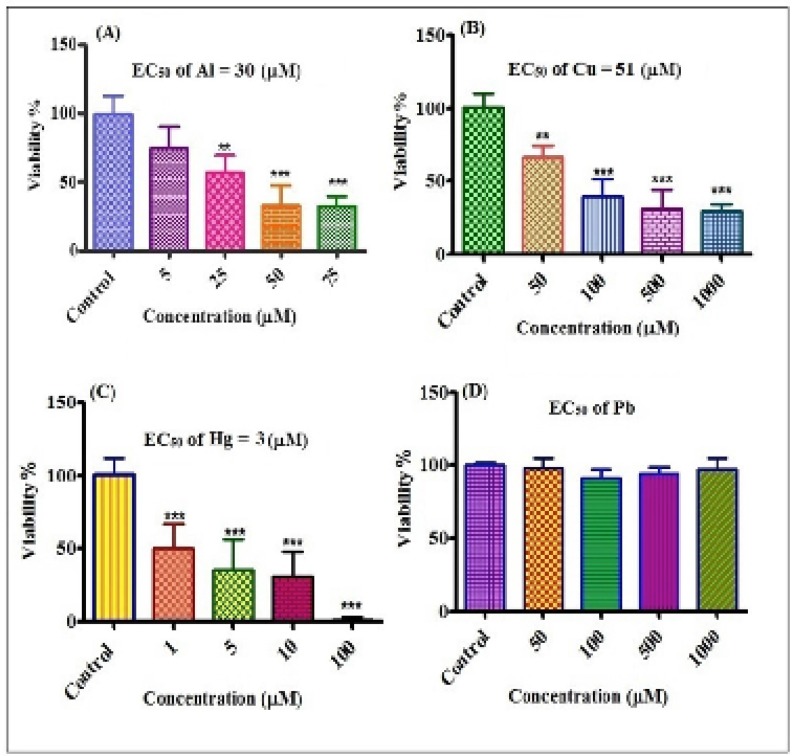
Cytotoxicity of different concentrations of Al^3+^, Cu^2+^, Hg^2+ ^and Pb^2+^on human lymphocytes. Viability of lymphocytes after treatment with Al^3+^(A), Cu^2+^(B), Hg^2+^(C), Pb^2+^(D) for 12 h. **P<0.01 and ***P<0.001

**Figure 3:**
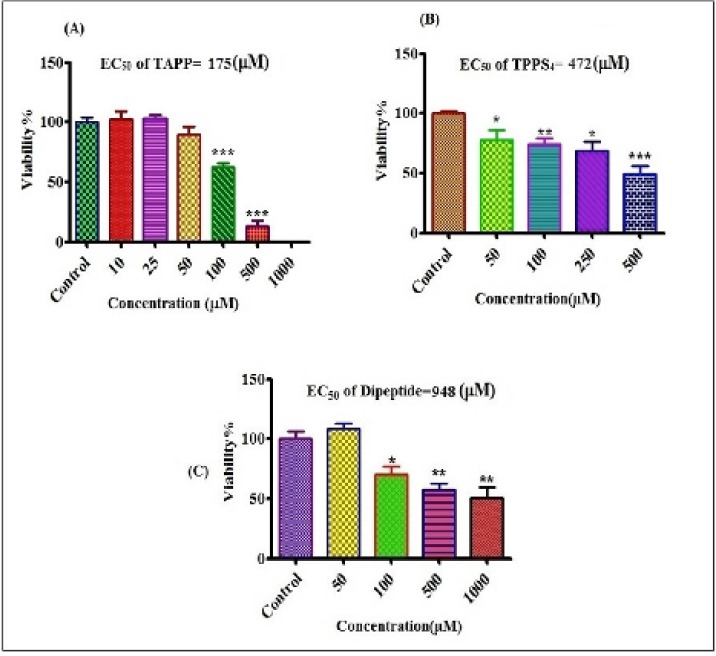
Cytotoxicity of different concentrations of TAPP, TPPS_4 _and dipeptide on human lymphocytes. Viability of lymphocytes after treatment with TAPP (A) TPPS_4 _(B) and dipeptide (C) for 12 h.*P<0.05, **P<0.01 and ***P<0.001

**Figure 4:**
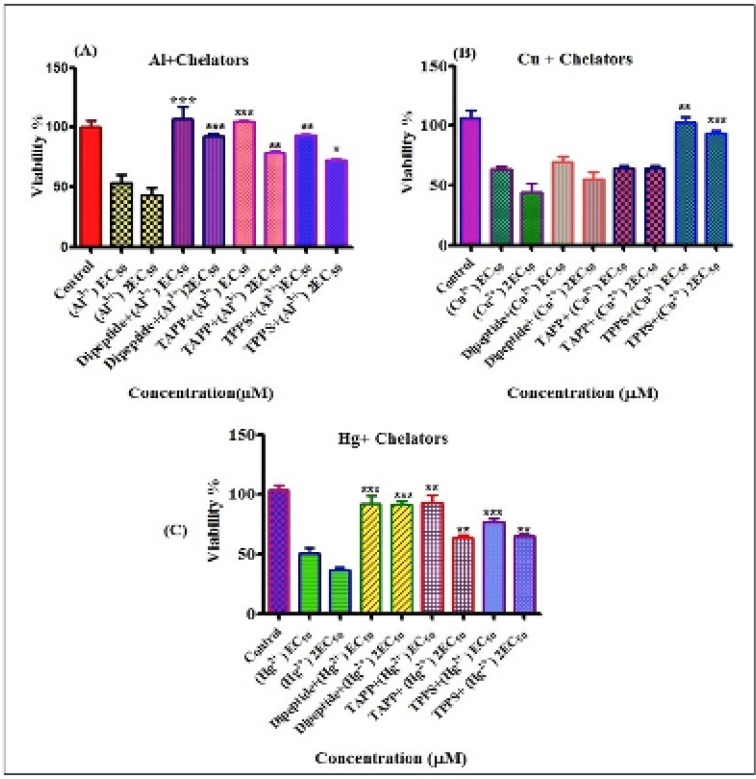
Compersion of protective effects of chelatorson cytotoxicity of toxicmetals.Protective effects of different chelators on cytotoxicity induced by EC_50 _and 2EC_50_of Al^3+^ (A), Cu^2+^ (B) and Hg^2+ ^(C) on human lymphocytes.*P<0.05, **P<0.01 and ***P<0.001

*Characterization of synthesised meso-tetrakis(4-trimethylanilinium)porphyrin (TAPP)*

The synthesis of TAPP approved using UV-Visible spectra, λ_max_= 416 nm (Soret band), 518, 556, 598, and 636 nm (Q bands); FT- IR spectra (KBr, cm^-1^**),** ν_max_:1118 (C-N), 1342 (C=C exocyclic pyrrol) 1471 (C=Cendocyclicpyrrol), 1606 (C=N), 2800-2900 (C-H methyl, C-H aliphatic & aromatic), 3000-3600 (N-Hpyrrol,N-anilinium); ¹H-NMR spectra (300 MHz, D_2_O, δ) 8.68 (s, N-H pyrrol) 8.07, 8.11(d, phenyl) 3.80 (s, CH_3_). For all above data, please refer to the supplementary information

*Characterization of synthesised Tetrakis(4-sulfonatophenyl)porphyr in(TPPS*_4_ *)*

The synthesis of (TPPS_4 ) _approved using UV-Visible spectra, λ_max_= 412 nm (Soret band) 512, 550, 578 and 632 nm (Q bands). FT- IR spectra (KBr, cm^-1^**)** ν_max_:1000-1380 (=C-N, S=O) 1336 (C=C exocyclic pyrrol) 1382 (C=C endocyclicpyrrol) 1560 (-C=N), 2800-3010 (C-H aliphatic & aromatic) 3000-3600 (N-H pyrrol,O-H).¹H-NMR spectra (300 MHz, D_2_O, δ): 8.12 (tetraphenyl), 7.63 (tetrapyrrol) For all above data, please refer to the supplementary information.

*Kinetic study of chelating reactions of dipeptide,TAPP and TPPS*_4_

The UV-Visible absorbance spectra was investigated to study the kinetic reactions of synthesized chelators [Histidine*-β*-Alanine (~10^-5^M), TAPP (~10^-5^M) and TPPS_4_ (~10^-5^M)] with different concentrations (~10^-1^ to ~10^-5^M) of metalions (Al^3+^, Cu^2+^, Hg^2+^ and Pb^2+^). Interactions of dipeptide, TAPP and TPPS_4_ with various concentrations of metal ions show that, the maximum absorption band of dipeptide (214 nm) TAPP (416 nm) and TPPS_4 _(412) reduced and shifted to short and longer wavelength in respect to the size of metal ions.we observed significant changes on the four Q bands in the visible region in such that they reduced to two bands due to the structural symmetry increase from C_2v_ to D_4h_ point groups (38). The results show at initial times (about 5-10 minutes) chelating with metals, which have same concentration with chelators (10^-5^M) were faster, but these reactions of metals with concentrations higher than chelators were slower. To compare the chelating ability between dipeptide and porphyrins the optimal concentration of metals was found to be~10^-3^ M for all chelators. In terms of rate of reaction, the experimental process of chelating shows that the reactions are complited at the first ten minutes, in this regard based on the rate law the reactions was found to be first order. using the integrated first order rate law of Ln [A]= kt+ Ln[A_0_] the rate constant (*k*) was determined from the plot of -Ln [A/A_0_] vs. time which gives a straight line with a slope of* k*. In Figure 1,it is shown that the order of rate constant increase for (Al^3+^) is such *k*_dipeptide_>*k*_ TAPP_>*k*_TPPS4_, which indicates that the reaction of dipeptide with Al^3+^ is faster than TAPP and TPPS_4_. The results of rate order constant increase for other metal ions, Cu^2+^, Pb^2+^, Hg^2+^ are:(Cu^2+^) *k*_TPPS4_>*k*_TAPP_>*k*_ dipeptide_, (Pb^2+^) *k*_dipeptide_>*k*_ TPPS4_>*k*_ TAPP _and (Hg^2+^) *k*_TAPP_>*k*_TPPS4_>*k*_ dipeptide_, these observations and calculated data represent that all of the chelators have high chelating potential with these metal ions. Histidine-*β*-Alanine is a multidentate ligand with five potential metal-coordinating sites (two N of imidazole ring, one carboxylate group, an amide linkage and a terminal amino group). From the structural study survey (39) two types of structure was introduced, the tetrahedral and octahedral complexes. About the porphyrin chelators the direct coordination between metal ions and tetrapyrrole-core also somewhat extends the conjugation from porphyrin to metal ions. According to quantum theory, an electronic excitation involved in a larger conjugated system requires lower energy absorption, corresponding to lower radiation frequency or longer wavelength (40). but the accurate configuration and chelating ability of chelators can depend on size of the metal cation, ligand-to-metal ratios, the ionic strength of the supporting solution, structure of chelator and charge density of a metal ion (39). The Results indicate that for Al^3+^and Pb^2+^, rate constants of dipeptide is relatively higher than porphyrin derivets and for Cu^2+^ and Hg^2+^, porphyrins show maxiumun chelating rate.

*In-Vitro assay of cytotoxicity induced by metals, chelators and metal-chelatorcomplexes*

The effects of different concentrations of metals on human lymphocyte viability were shown in Figure 2. EC_50_ values calculated using prism software were 30, 51 and 3 µM for Al^3+^, Cu^2+^ and Hg^2+ ^respectively and for Pb^2+^ no cytotoxicity was observed on lymphocyte cells up to 1000 µM concenteration. The order of the cytotoxicity of metals on lymphocyte was: Hg^2+^>Al^3+^>Cu^2+^>Pb^2+^.

Evaluation of chelatorʹs effects on cytotoxicity was shown in Figure 3. EC_50 _measured for dipeptide, TPPS_4_ and TAPP were equal to 948, 472 and 175 µM, respectively.The order of the cytotoxicity of chelators was: TAPP>TPPS_4_>dipeptide.

Preventing effects of chelators against cytotoxicty of metals were shown in Figure 4 Our results show that all of chelatorsr educeed cytotoxicity of metals on lymphocytes. Compersion of the chelators at preventing toxic metal induced cytotoxicity on human lymphocytes were: For Al^3+^(30 µM) dipeptide>TAPP>TPPS_4_ and For Al^3+^ (60 µM) dipeptide>TAPP>TPPS_4_. Based on the letrature servey, the toxic preventing effect of dipeptide relates to the antioxidant properties of this compound (41). For Cu^2+^ (51 µM) TPPS_4_>dipeptide~TAPP and For Cu^2+^(102 µM) TPPS_4_>TAPP>dipeptide. For Hg^2+^ (3 µM) dipeptide~TAPP>TPPS_4_and For Hg^2+^(6 µM) dipeptide>TAPP~TPPS_4_.

The data obtained from the kinetic studies represent that order of rate constant of Al^3+^with chelators are: dipeptide>porphyrins, because Al^3+ ^has the smallest atomic radius and the highest charge among other 3 cations, makes it capable for accepting pair electrons from N and O atoms of dipeptide (39). For Cu^2+^ metal ion, the order of rate constants is: TPPS_4_>TAPP>dipeptide Which can be related to coordination of metal ion to tetrapyrrole-core in TPPS_4_ that typically accept sp^3^d^2^ hybrid with four orbitals in porphyrin plane and two orbitals in vertical ± z direction, cordinated with two water molecule giving rise to octahedral geometry. However, Cu^2+^ cation may experience the so-called “Jahn-Teller effect” because of its asymmetrical d-electron configuration, which results in geometry distortion and extra binding strength (40). The generated complex between Cu^2+^ and dipeptide is such that two moelcules of dipeptide with one molecule of water makes it to be five coordination complex. The four nearest ligand atoms are the terminal amino nitrogen, the amide nitrogen, a carboxylate oxygen of one of the dipeptide molecules, and the nitrogen of the imidazole from the sec molecule. this shows that each dipeptide molecule has potential of bounding to two different Cu^2+^cation centers (20). For Hg^2+^cation coordination with chelators, rate order constants are TAPP>TPPS_4_>dipeptide by this order the monomeric structure of Hg^2+^ complexes with dipeptide shows the lower stability, we assume that the complex can possesses a polymeric structure [HgLH]_n_^2+^. In this regard the mercury complexation with dipeptide reverses the tautomeric preference between protonation of the N-atoms of the imidazole ring (20). The rate constants order for Pb^2+^cation complexation with ligans are: dipeptide>porphyrins, because Pb^2+^ has lower ratio of charge to radius, therefore can be easily coordinated to dipeptide potential coordinating sites. The strength of porphyrin-metal ion interaction may depend on different factors, including the porphyrin substitutions, charge state (such as H_2_P or P^2−^H_4_P^2+^) and type of metal ion, the factors may be more than these but in this work, some of them considered as an effective factor. In supporting of this discussion refrence (4) has useful information regarding to the binding of N-alkylated porphyrin HN-Me-TPPS with cations Cd^2+^ and Zn^2+^shows the following rate constants:1.3×10^−2^ and 3.3×10^-1^, respectively, this is exactly what we observed during the experimental procedure. The binding trends revealed in these quantitative data are consistent with our qualitative predictions, although in comparing with some literature surveys, there might be some diffrences, this is unavoidable because they might use basically different materials and experimental methods. The findings *in-vitro* on human lymphocytes suggested that greater concenterations than EC_50_ of metal ions, Pb^2+^ (1000 µM), Cu^2+^ (51 µM) Al^3+^(30 µM) and Hg^2+^ (3 µM) can decrese Viability% of lymphocytes. For comparing chelators effects against cytotoxicity of metals, we treated lymphocytes with EC_50_ and 2EC_50 _values of metals. The results indicated that chelators reduced toxicity of metals against human lymphocytes. Regarding the Pb^2+^ no cytotoxicity was determined on lymphocytes up to 1000 µM concenteration. The lymphocyte cytotoxicity with Pb^2+^ chelator complexes were even lower than Pb^2+^ (data not shown). The difference between chelators effects against cytotoxicity of metals can correspond with reasons that have been described about kinetic study. Positive results about protective effects of dipeptide (his-*β*-alanine) and two porphyrin derivatives on metal cytotoxicity toward human lymphocytes and numerous studies that have been done by many researches, acknowledge the importance of these compounds in drug developments. his-*β*-alanine, a naturally occurring di-peptide, is present in large amounts in long-lived human tissues. Numerous evidence have indicated the multi-functionality of this dipeptide in the human body (4,34) such as physiological buffering agent and a metal ion (e.g., zinc and copper) chelator, regulator of the amount of transition metal ions in biological fluids and tissues. Besides, it has ability to form complexes with metal ions (42-45). his-*β*-alaninehas been used as an eye-drop component used for the treatment and inhibition of senile cataract (46). In addition, his-*β*-alanine has been shown to suppress the accumulation of amyloid beta peptide in the central nervous system of transgenic mouse model for Alzheimer’s disease (47). and attenuate the *in-vitro* fibrillogenesis of amyloid beta peptide (1–44) (48). It is a potent scavenger of both reactive oxygen species (ROS) and reactive nitrogen species (RNS) which excite peroxidation of unsaturated lipids present in membranes as well as of toxic reactive α, β-unsaturated aldehydes deriving from this oxidation (49,50). Possessing anti-aging functions (51,52), his-*β*-alanine illustrated a well-documented anti-glycating activity in proteins, including low-density lipoproteins, glucose decline products, esterase, and histones (53-54). his-*β*-alanine was reported to increase the thermal unfolding and water availability of glycated protein species (55). It also mitigates and/or prevents the variation in electrophoretic dynamismoperated by glyceraldehyde 3-phosphate (56). A study in 2006 by Mahmood* et al*. showed that Zn and the antioxidant his-*β*-alaninecan stabilize the entirety of the small bowel and motivate repair processes in the gut, both *in- vitro* and *vivo* models (57). It has been reported that Zn and his-*β*-alaninemay induce anti-oxidative stress enzymes *in-vitro* and *in-vivo* model (58). One of the long-standing goals of both researchers and oncologists is to create a framework for the complete cure of cancer with less toxic effect and make better Quality of life (QOL). Experiments to test bioactivation of neutral reagents by light led to modern photodynamic therapy. The priority of photodynamic therapy has no offense during treatment and the selective agglomeration of photosensitizers in tumor tissue. Porphyrin and its analogues are the most effective photosensitizers for PDT. These photosensitizers have a strong absorption band (ε5×10^5^M^−1^cm^−1^) around 400 nm called the Soret band and weak absorption bands between 500 and 800 nm named Q bands. Despite the large molar absorption coefficient, the Soret band is not adequate for PDT of deeper tumor tissues. The Q1 band (600–800 nm) is generally used for PDT (59-60). Anti-HIV-1 activity of the porphyrins was determined by multiple nuclear activation of galactosidase indicator cell (MAGI) assay. Cytotoxicity of different kind of porphyrins are determined. Interestingly, the cytotoxicity of the iron-(III) complexes was lower than that of the corresponding porphyrin free bases. A similar effect on the cytotoxicity was reported by Song *et al*. The introduction of the iron atom might improve the selectivity of the binding of the porphyrin to the V3 loop region. The low cytotoxicity and highanti- HIV-1 activity of these iron(III) porphyrins are a large benefit to the usage of these metal complexes (62). Our results also showed that pretreatment of lymphocytes with chelators clearly indicate that all of the chelators reduce toxicity of the metals on lymphocytes. Viability % of human lymphcyts following addition of dipeptide was higher than those of porphyrin derivetives.

## Conclusions

In this work, synthesise of dipeptide (his-*β*-alanine) was done by solution phase peptide synthesis (SPS) and solid phase peptide synthesis (SPPS) on Wang resin. study of chelating properties of dipeptide (his-*β*-alanine) and porphyrin derivatives by UV-Visible absorbance spectra revealed that chelating activity depends on concentration of metal, physicochemical properties of metaland finally molar ratio of metal and chelator, The order of the chelator strength at adsorbing Al^3+^(10^-3^M) and Pb^2+^ (10^-3 ^M) was:dipeptide> porphyrin compounds. It is propebly due to *k*_dipeptide_>*k*_ porphyrin_. For Cu^2+ ^(10^-3^M) and Hg^2+ ^(10^-3 ^M): *k*_porphyrin _>*k*_ dipeptide_. Results of pretreatment of lymphocytes with chelators clearly indicate that all of the chelators reduce toxicity of the metals on lymphocytes. viability% of human lymphcyts following addition of dipeptide was higher than those of porphyrin derivetives.
